# Comparative effect of Kegel and Pelvic rocking exercises on primary dysmenorrhea: A quasi-experimental study

**DOI:** 10.6026/973206300213531

**Published:** 2025-10-31

**Authors:** Patel Divyanka Navinbhai, Mahalakshmi B, Siva Subramanian N

**Affiliations:** 1 Department of Obstetrics and Gynecological Nursing, Nootan College of Nursing, Sankalchand Patel University, Visnagar, Gujarat - 384315, India; 2 Department of Pediatric Nursing, Nootan College of Nursing, Sankalchand Patel University, Visnagar, Gujarat - 384315, India; 3 Department of Psychiatric Nursing, Nootan College of Nursing, Sankalchand Patel University, Visnagar, Gujarat - 384315, India

**Keywords:** Primary dysmenorrhea, kegel exercises, pelvic rocking exercises, menstrual pain, pelvic floor strengthening, adolescent girls

## Abstract

Primary dysmenorrhea is a common cause of menstrual pain among adolescents, affecting daily activities and quality of life. Our quasi-experimental study assessed 300 adolescent girls, comparing the effect of Kegel exercises (n=150) and pelvic rocking exercises (n=150) over four menstrual cycles. Pain intensity was measured using the Numerical Pain Rating Scale before and after the intervention. Both groups showed statistically significant reductions in pain scores (p<0.001), with greater mean reduction in the Kegel group (2.59 ± 0.84) compared to the pelvic rocking group (1.55 ± 0.78). Categorical analysis revealed a higher shift from severe to mild pain among participants performing Kegel exercises. Thus, we show that pelvic floor strengthening offers superior pain relief and should be incorporated into adolescent health programs to reduce analgesic use and improve quality of life.

## Background:

Primary dysmenorrhea-menstrual pain in the absence of pelvic pathology-is one of the most common gynecological complaints among adolescent girls and young women, often causing significant discomfort, loss of productivity and reduced quality of life [1]. The prevalence of dysmenorrhea worldwide is estimated to be between 50-90% among menstruating females, with a substantial proportion experiencing moderate to severe pain [2]. Pain in dysmenorrhea is believed to arise from uterine ischemia secondary to excessive prostaglandin production, leading to intense contractions [3]. Non-pharmacological interventions, including physical exercises that target muscle strength, relaxation, or alignment, are increasingly evaluated for their potential to reduce pain without the adverse effects of medications [4]. Among such interventions, pelvic floor muscle exercises (often called Kegel exercises) have been shown to improve symptoms of primary dysmenorrhea [5]. Similarly, a recent systematic review and network meta-analysis (29 RCTs, ~1808 participants) demonstrated that various exercise interventions-including strength training, aerobic exercise, yoga, relaxation and the Kegel maneuver-were effective in reducing menstrual pain after eight weeks of intervention [6]. However, the evidence comparing pelvic floor strengthening (Kegel) specifically against other simple exercise modalities like pelvic rocking in adolescent populations remains limited [7]. A quasi-experimental study in Jordan comparing Kegel versus pelvic rocking exercises among adolescents showed greater pain reduction in the Kegel group over an eight-week period [8]. Therefore, it is of interest to describe the comparative effect of Kegel exercises and pelvic rocking exercises on menstrual pain intensity among adolescent girls.

## Methodology:

## Research design:

A quasi-experimental pre-test post-test control group design was employed to evaluate the effect of pelvic floor strengthening (Kegel) and pelvic rocking exercises on menstrual pain among adolescent girls.

## Setting and Population:

The study was conducted in selected rural areas of Dadra and Nagar Haveli. The population comprised adolescent girls aged 13-18 years experiencing primary dysmenorrhea with a baseline NPRS pain score ≥4.

## Sample and Sampling technique:

A total of 300 participants were recruited using purposive sampling, with 150 in the Kegel group and 150 in the pelvic rocking group. Girls with secondary dysmenorrhea, pelvic pathology, or recent hormonal/analgesic use were excluded.

## Ethical considerations:

Institutional Ethical Committee approval was obtained prior to data collection. Written informed consent from participants and parental/guardian assent were secured.

## Intervention:

Participants in the Kegel group performed pelvic floor muscle contractions for 5 seconds followed by 5 seconds relaxation, repeated 10-15 times per session twice daily, whereas the pelvic rocking group performed rhythmic anterior-posterior and lateral tilting of the pelvis in supine position for 10 minutes twice daily. Both interventions continued for four consecutive menstrual cycles with adherence monitored using participant-maintained exercise logs and weekly investigator supervision.

## Outcome measure:

Pain intensity was assessed using the Numerical Pain Rating Scale (0-10), recorded once during the pre-test (baseline) and again during the post-test after completion of the intervention.

## Data analysis:

Data were analyzed using SPSS (version XX). Descriptive statistics summarized demographic characteristics. Paired t-test evaluated pre-test and post-test changes within each group and independent t-test compared mean pain reduction between groups. Statistical significance was set at p < 0.05.

## Results:

Table 1 (see PDF) shows that the majority of adolescent girls in both groups was aged 14-15 years, Hindu by religion and belonged to nuclear families. Most were from rural areas, followed a vegetarian diet and had a weight range of 36-45 kg with adequate sleep hours and no regular physical exercise. Table 2 (see PDF) presents the pre- and post-test mean pain scores, demonstrating a statistically significant reduction in pain after intervention in both groups (p < 0.001), with a larger mean reduction observed in the Kegel group (2.59) compared to the Pelvic Rocking group (1.55). Figure 1 (see PDF) further illustrates the categorical distribution of pain severity, where the number of participants with severe pain dropped from 61 to 24 in the Kegel group and from 67 to 36 in the Pelvic Rocking group, with a corresponding rise in mild pain cases, indicating a clear shift toward lower pain intensity after the intervention. Together, these results confirm that both Kegel and Pelvic Rocking exercises were effective in reducing menstrual pain, with Kegel exercises producing a more pronounced improvement.

## Discussion:

Our study shows that both Kegel exercises (pelvic floor strengthening) and pelvic rocking exercises significantly reduced pain intensity among adolescent girls with primary dysmenorrhea. The mean pain score decreased more in the Kegel group (mean difference = 2.59) compared to the pelvic rocking group (mean difference = 1.55). Categorical analysis also revealed a greater shift from severe to mild pain in the Kegel group. These results support the hypothesis that targeted pelvic floor strengthening is more effective in relieving dysmenorrhea pain than simple pelvic mobilization exercises. Our findings are strongly supported by previous systematic reviews and meta-analyses. Noetel *et al.* (2024) conducted a network meta-analysis of randomized controlled trials (RCTs) and concluded that all exercise modalities-relaxation, strength training, aerobic, yoga, mixed exercise and Kegel maneuvers-were effective, with relaxation and strength-based exercises showing the largest effect sizes [9]. Matthewman *et al.* (2018) reported that exercise interventions significantly reduced both pain intensity (-1.89 cm VAS) and pain duration (-3.86 h) compared with controls, highlighting exercise as a viable first-line management option [10]. Cai *et al.* (2025) further demonstrated a dose-response relationship, where aerobic exercise programs performed more than 3 times per week had a superior effect on pain intensity reduction [11]. Our study's finding that Kegel exercises produced a greater reduction in pain compared to pelvic rocking aligns with evidence from Giordani *et al.* (2022) who compared manual therapy (MT), pelvic floor exercises (PFE) and their combination, reporting that all interventions were effective but MT + PFE had the strongest impact [12]. Zheng *et al.* (2024) confirmed that resistance and pelvic floor exercises were among the most effective when performed consistently for over 8 weeks [13]. Öz *et al.* (2025) compared resistance training and stretching exercises and found both effective, with resistance training producing slightly greater improvements in quality of life [14]. Pelvic rocking exercises were also effective in our study, though to a lesser extent. Similar findings were reported by Saikia *et al.* (2024) who observed significant reductions in pain scores among adolescent girls following pelvic rocking interventions [15]. This suggests that even simple pelvic mobilization can provide pain relief and may be a useful alternative when pelvic floor instruction is not feasible. Samy *et al.* (2019) also demonstrated that rhythmic exercises like Zumba reduced both pain intensity and duration significantly after 4 and 8 weeks [16].

The physiological explanation for our results may lie in improved pelvic blood flow and reduced uterine ischemia. jaleel *et al.* (2022) showed that aerobic exercise decreases prostaglandin synthesis and alleviates uterine muscle spasm, thereby reducing pain [17]. Exercise also triggers the release of beta-endorphins and enkephalins, which modulate nociceptive transmission [18], [19]. Sluka *et al.* (2018) highlighted in their Cochrane review that endorphin release is one of the principal mechanisms behind exercise-induced analgesia [20]. Our findings are consistent with Yadav *et al.* (2024) who demonstrated that combined exercise training involving deep breathing, Kegel and core exercises reduced dysmenorrhea pain significantly when performed in both follicular and luteal phases [21]. Koçoglu *et al.* (2025) showed that combining pelvic floor training with relaxation therapy produced improvements in functional and emotional pain scores [22]. Similarly, Ortiz *et al.* (2015) implemented a multi-component physiotherapy program including strengthening, stretching and relaxation and jogging, which significantly reduced VAS pain scores [23]. Multiple systematic reviews have confirmed the role of exercise and physiotherapy. Fuentes-Aparicio *et al.* (2023) concluded that therapeutic exercise produces clinically meaningful improvements in pain intensity and duration [24]. Sahin (2017) similarly reported significant pain reduction with physiotherapy techniques despite variability in study quality [25]. Duncun *et al.* (2017) in their systematic review of complementary therapies also found exercise to be one of the most effective non-pharmacologic interventions [26]. Several RCTs have focused on yoga and stretching, which can be compared with our results. Aksu *et al.* (2025) demonstrated that yoga poses significantly decreased pain severity among nursing students and Kanchibhotla (2023) found that yoga reduced both pain intensity and duration over three consecutive cycles [27-28]. Although our study did not include a yoga intervention, these findings highlight that both strengthening and relaxation exercises may be complementary. Our study adds value by focusing exclusively on pain, using a structured protocol for both Kegel and pelvic rocking exercises and targeting adolescent girls-a population often underrepresented in clinical trials. Future research should include randomized controlled designs, longer follow-up and biochemical markers such as prostaglandin levels and combined interventions including yoga, relaxation and aerobic components to assess synergistic effects.
